# Altered neonatal cord blood oxylipidome in association with exposure to particulate matter in the early life environment

**DOI:** 10.1186/2049-3258-73-S1-O5

**Published:** 2015-09-17

**Authors:** Dries Martens, Sandra Gouveia-Figueira, Narjes Madhloum, Bram G Janssen, Michelle Plusquin, Bertil Forsberg, Malin L Nording, Tim S Nawrot

**Affiliations:** 1 Hasselt University, Diepenbeek, Belgium; 2 Department of Chemistry, Umeå University, Umeå, Sweden; 3 Centre for Environmental Sciences, Hasselt University, Diepenbeek, Belgium; 4 Division of Occupational and Environmental Medicine, Umeå University, Umeå, Sweden

## Background and aims

As part of the lipidome, oxylipins are bioactive lipid compounds originating from oxidation of different fatty acids. We studied oxylipin profiles in cord blood as part of the lipidome in early life in association with *in utero* exposure to particulate air pollution.

## Methods

Oxylipins were extracted from 197 cord blood plasma samples from the ENVIR*ON*AGE (ENVIRonmental influence *ON* AGEing in early life) birth cohort in Belgium. Thirty-seven specific oxylipins reflecting the cyclooxygenase (COX), lipoxygenase (5-LOX and 12/15-LOX) and cytochrome P450 (CYP) metabolic pathways were quantified by ultra-performance liquid chromatography coupled to electrospray ionization tandem mass spectrometry (UPLC-ESI-MS/MS). Principal component analysis and multiple regression models were applied to associate oxylipin pathways as well as individual metabolites with *in utero* PM_2.5_ exposure, while adjusting for newborns gender, gestational duration, maternal age, maternal smoking status, maternal BMI, and cord blood total cholesterol and HDL levels.

## Results

PM_2.5_ exposure during pregnancy averaged (25th-75th percentile) 15.7 (13.5-17.5) μg/m^3^. Six metabolites combined in a principal component (PC), representing the 5-LOX pathway was positively associated with PM_2.5_ exposure during the entire (? = 0.11; 95% CI: 0.03, 0.20; p = 0.01) and second trimester of pregnancy (β = 0.06; 95% CI: 0.01, 0.10; p = 0.01). Eleven metabolites combined in a PC representing the 12/15-LOX pathway was positively associated with PM_2.5_ during the second trimester of pregnancy (β = 0.09; 95% CI: 0.03, 0.15; p = 0.006). No associations were found for the COX and CYP pathways.

## Conclusions

*In utero* exposure to particulate matter was associated with the lipoxygenase pathways (5-LOX and 15-LOX) in newborns. These changes at the level of the lipidome indicate an altered inflammatory state of the newborn at birth induced by air pollution during *in utero* life.

